# Balance and Risk of Fall in Individuals with Bilateral Mild and Moderate Knee Osteoarthritis

**DOI:** 10.1371/journal.pone.0092270

**Published:** 2014-03-18

**Authors:** Nafiseh Khalaj, Noor Azuan Abu Osman, Abdul Halim Mokhtar, Mahboobeh Mehdikhani, Wan Abu Bakar Wan Abas

**Affiliations:** 1 Department of Biomedical Engineering, Faculty of Engineering, University of Malaya, Kuala Lumpur, Malaysia; 2 Department of Sport Medicine, Faculty of Medicine, University of Malaya, Kuala Lumpur, Malaysia; West Virginia University School of Medicine, United States of America

## Abstract

Balance is essential for mobility and performing activities of daily living. People with knee osteoarthritis display impairment in knee joint proprioception. Thus, the aim of this study was to evaluate balance and risk of fall in individuals with bilateral mild and moderate knee osteoarthritis. Sixty subjects aged between 50 and 70 years volunteered in this study. They were categorized into three groups which were healthy (n = 20), mild (n = 20) and moderate (n = 20) bilateral knee osteoarthritis groups. Dynamic and static balance and risk of fall were assessed using Biodex Stability System. In addition, Timed Up and Go test was used as a clinical test for balance. Results of this study illustrated that there were significant differences in balance (dynamic and static) and risk of fall between three groups. In addition, the main (most significant) difference was found to be between healthy group and moderate group. Furthermore, on clinical scoring of balance, the “Timed Up and Go” test, all three groups showed significant difference. In conclusion, bilateral knee osteoarthritis impaired the balance and increased the risk of fall, particularly in people with moderate knee osteoarthritis.

## Introduction

Osteoarthritis (OA) is a degenerative and progressive joint disease that mainly involves weight-bearing joints such as the hip, knee, and ankle. It is considered as one of the leading causes of lower limb disabilities among the elderly [1]. Degenerative OA of the knee is one of the most common forms of osteoarthritis worldwide. It causes major loss of function [2]–[4] and activity limitations [5] as well as posing a considerable socioeconomic burden on the societies and families due to disabilities [1], [4], [6]. Knee OA results in progressive loss of function including: gait, stair climbing and other physical activities which involve lower limb. In fact, it reduces the quality of life.

People with knee OA experience loss of proprioception [5], [7]–[9], which may affect postural stability and risk of fall. Postural stability could be defined as control over body's position in space for orientation and balance purpose [10]. It is essential for us to maintain postural stability (static and dynamic balance) during activities of daily living (ADLs) and ambulation. Impaired postural stability is one of the main reasons of falls in older adults and thus constitutes a significant public health problem [11], and it is considered as one of the leading causes of fatalities and hospital admissions [12].

Proper balance is essential for maintaining postural stability while performing functional activities and for fall avoidance [13]. Balance (dynamic and static) is a complex function which requires integration of sensory information regarding the position of the body and the ability to make appropriate motor response to body movement [6], [14]. More precisely, it depends on sensory inputs from somatosensory (proprioception), visual and vestibular systems [4], [7]; as well as, response of muscles. Static balance refers to maintaining equilibrium while standing in one spot; whereas, dynamic balance involves motion and is defined as maintaining equilibrium during locomotion [15].

Falls and loss of balance most commonly occur during movement-related tasks such as walking and less frequently during static activities [4]. Previous studies assessed postural stability (balance) and risk of fall among individuals with knee OA using different methods and evaluation protocols. Some studies used functional tests such as “functional reach” [6] and “Step test” [4], [6], and some used technology-based balance systems such as “Balance Master- NeuroCom” [6], [16] and “Biodex Stability System (BSS)” [11], [17]. Studies which used postural sway for assessing balance reported that people with knee OA displayed greater postural sway compared to their healthy controls [4], [8], [18]–[20]. A comprehensive balance assessment including measures of both static and dynamic balance using both technology-based system and clinical test among bilateral knee OA patients is required. Illustrating the level of balance and risk of fall in individuals with knee OA is essential, particularly for rehabilitation purposes. Objective assessments as baseline and progression data will enable the rehabilitation team to set and customize goals, modify and evaluate their work. Thus, the aim of this study was to evaluate postural stability and risk of fall in individuals with bilateral mild and moderate knee OA using the Biodex Balance System and the “Timed Up and Go” test (TUG). In addition, this study aimed to determine whether OA severity grade would affect postural stability and risk of fall.

## Methods

### Ethical statement

This study was approved by Medical Ethic Committee in University Malaya Medical Centre (UMMC) (919.18/May2012). All participants read and signed a written consent form.

### Participants

Forty subjects with bilateral mild and moderate knee OA (each group 20 participants) and 20 aged matched healthy controls participated in this study. Participants with knee OA were referred from UMMC, Kuala Lumpur, Malaysia; and healthy controls were recruited through advertisement. All the participants underwent clinical assessment which was done by a medical doctor, and diagnosis of knee OA was confirmed by X-ray (extension position); which were graded by two specialists (radiology and sport medicine) using Kellgren-Lawrence grading scale. According to Kellgren-Lawrence scale, grade 0 refers to the knees with no features of osteoarthritis and grade one is doubtful knee osteoarthritis, in which narrowing of the joint space and possible osteophytes can be seen. Grade two is mild knee OA, with small osteophytes and possible narrowing of the joint. Grade three is known as moderate knee OA, which is characterized by multiple, moderately sized osteophytes, definite joint space narrowing, and possible deformation of bone ends. Lastly, severe knee OA is classified as grade four which is presented by multiple large osteophytes, severe joint space narrowing, marked sclerosis and definite bony end deformity.

Participants with knee OA were included if their age were between 50 and 70 years, had bilateral mild knee OA (grade II) and moderate knee OA (grade III), and were independent in activities of daily living (ADLs). Exclusion criteria included lower limb joint replacement, knee surgery for the past 12 months, any lower limb fractures during the past six months, intra-articular injection in the previous 6 months, neurological disorders, diabetes mellitus, history of recent fall (past 12 months), underwent treatments such as rehabilitation programs (physiotherapy and hydrotherapy) and supplements (e.g. glucosamine) and any other condition that might impair balance.

Participants were categorized into three groups: healthy (without knee OA), mild knee OA (knee OA grade II), and moderate knee OA (knee OA grade III). Anthropometric data of all the participants were obtained prior to balance and risk of fall assessment.

### Protocol of postural stability and risk of fall assessment

Balance and risk of fall were assessed using the Biodex stability system and the “Timed Up and Go” test. The TUG is internationally accepted functional, dynamic test of balance with known reliability and validity, as well as being low cost and easy to apply [21]. The TUG test measures the time in seconds that takes a subject to stand up from a chair, walk three meters at a comfortable and safe pace, turn around, walk back to the chair and sit down [21]. Subjects with score less than 10 seconds are considered normal, less than 15 seconds are at risk of fall, less than 20 seconds are independent in ambulation and are able to climb the stairs, and greater than 30 seconds need help with chair, toilet and are not able to climb the stairs [19]. In this study, participants were asked to perform TUG three times; and the final TUG score was the average of three times.

Biodex Stability System (BSS; Biodex Medical System Inc., Shirley, NY, USA) is a balance device which was designed to assess and record balance and neuromuscular control under dynamic stress [21]. BSS is multiaxial device with an unstable balance platform, to measure postural stability under dynamic tests, which provides up to 20° surface tilt in a 360° range of motion. This platform is free to move about the anterior-posterior (AP) and medial-lateral (ML) axes simultaneously [22], which permits three measurements to be obtained including overall stability index (OSI), anterior-posterior stability index (APSI) and medial-lateral stability index (MLSI) [23]. The formulas for OSI, APSI and MLSI are shown below:
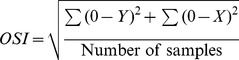





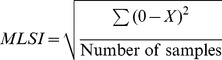





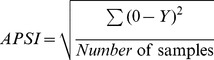
BSS provides 12 levels for assessing balance and risk of fall, level 12 is the most stable and level one is the most difficult task (unstable). The OSI is considered the most reliable indicator of postural stability; and Arnold and Schmitz (1998) suggested that the overall score is the tool that can be used to assess balance. Overall score can be affected by AP and ML scores. Lower overall scores indicate better balance and high score means poor balance. In addition, the risk of fall would only be presented by the actual score in the overall stability index.

Static balance, dynamic balance and risk of fall of all the participants were assessed using BSS. Participants were asked to step on the platform in a bipedal stance, with bare feet and eyes open [11] looking forward to monitor, while their hands are hanging by their sides (hand support was not permitted). They were asked to stand straight, not to change their feet position, and only sway their body when it was needed. The handles were available for safety purpose, but touching the handles would cancelled the trial.

Failure to control the positioning of the feet may significantly confound explanation of clinical or experimental balance measures [24]; in other words, foot placement can influence stabilizing reactions. However, positioning the feet outside of the subject's or patient's preferred (comfortable) stance position may affect the measured postural response [24]. Thus, measurements from two different feet positions were used to avoid any bias that may influence the results; one of them was defined (specific) position which was adopted according to the findings of McIlroy and Maki (1997), and was almost the same for all the participants. The defined (specific) position was based on the average absolute stance width for elderly at 0.16 (0.04) m (10.4% height) and average stance angle at 16.6 (11.3) degree [24]. The second feet position was the functional position assumed by the subjects on functional or comfortable standing position [17], [25]. Functional position is the standing position that they have during walking and other ADLS.

Balance and risk of fall were assessed using two trials over a period of 30 seconds with 10 seconds rest in between. Subjects were given 5 minutes rest between two testing positions to avoid the effect of fatigue and pain on the scores. Order of testing was chosen randomly. Assessing dynamic bilateral stance was done setting platform at level 8 [23]; for static level, platform was set to remain static with no tilt; and for assessing risk of fall, level 8 and level 6 to 2 was used. Prior to the testing, all the participants underwent a familiarization session. Test procedure was briefly explained, and participants underwent only one practice trial to get familiar with BSS and understand what we wanted them to do.

### Statistical analyses

The SPSS Statistics version 17.0 was used for all statistical analysis. The alpha level of 0.05 was defined as statistically significant for all the tests. One-way ANOVA (analysis of variance) Post-hoc (Tukey) was used to assess the difference between groups. Post-hoc was used to determine where the difference lies. In addition, descriptive analysis was used to assess mean and standard deviation (SD) of all the variables.

## Results

Total of 60 subjects participated in this study (20 male, 40 female). The age of participants ranged from 50 to 69 years. Table 1 demonstrates anthropometric information of all the participants; all the participants were aged-matched. Table 2 represents foot angle (degree) and heel width (cm) among three groups. Heel width is defined as the distance between the midlines of the two heels [24]. The feet angle was calculated between the lines joining the centre of the heel and the great toe of each foot [24].

**Table 1:** Anthropometric information of all the participants.

	Healthy	Mild knee OA	Moderate Knee OA
	Mean (SD)	Mean (SD)	Mean (SD)
Age (year)	58.45 (4.8)	57.1 (5.0)	59.9 (5.9)
Height (cm)	156.18 (7.3)	156.8 (6.2)	159.1 (10.2)
Weight (Kg)	64.9 (8.9)	66.2 (8.1)	70.8 (7.4)
BMI (Kg/m^2^)	26.6 (1.7)	26.8 (2.3)	28.0 (3.5)
Participants*	20 (M:8, F: 12)	20 (M:5, F: 15)	20 (M:7, F: 13)

*Number of subjects: M is male and F is female.

**Table 2:** Feet angle and heel width.

	Feet angle-degree		Heel width-cm	
	Mean (SD)		Mean (SD)	
	Defined	Functional	Defined	Functional
Normal	20.0	40.3 (12.9)	17.6 (1.9)	13.3 (5.0)
Mild group*	20.0	34.3 (11.3)	18.8 (1.0)	14.7 (3.3)
Moderate group*	20.0	38.4 (10.1)	18.1 (1.9)	14.1 (1.4)

*Severity of Knee OA.

**Figure 1:**
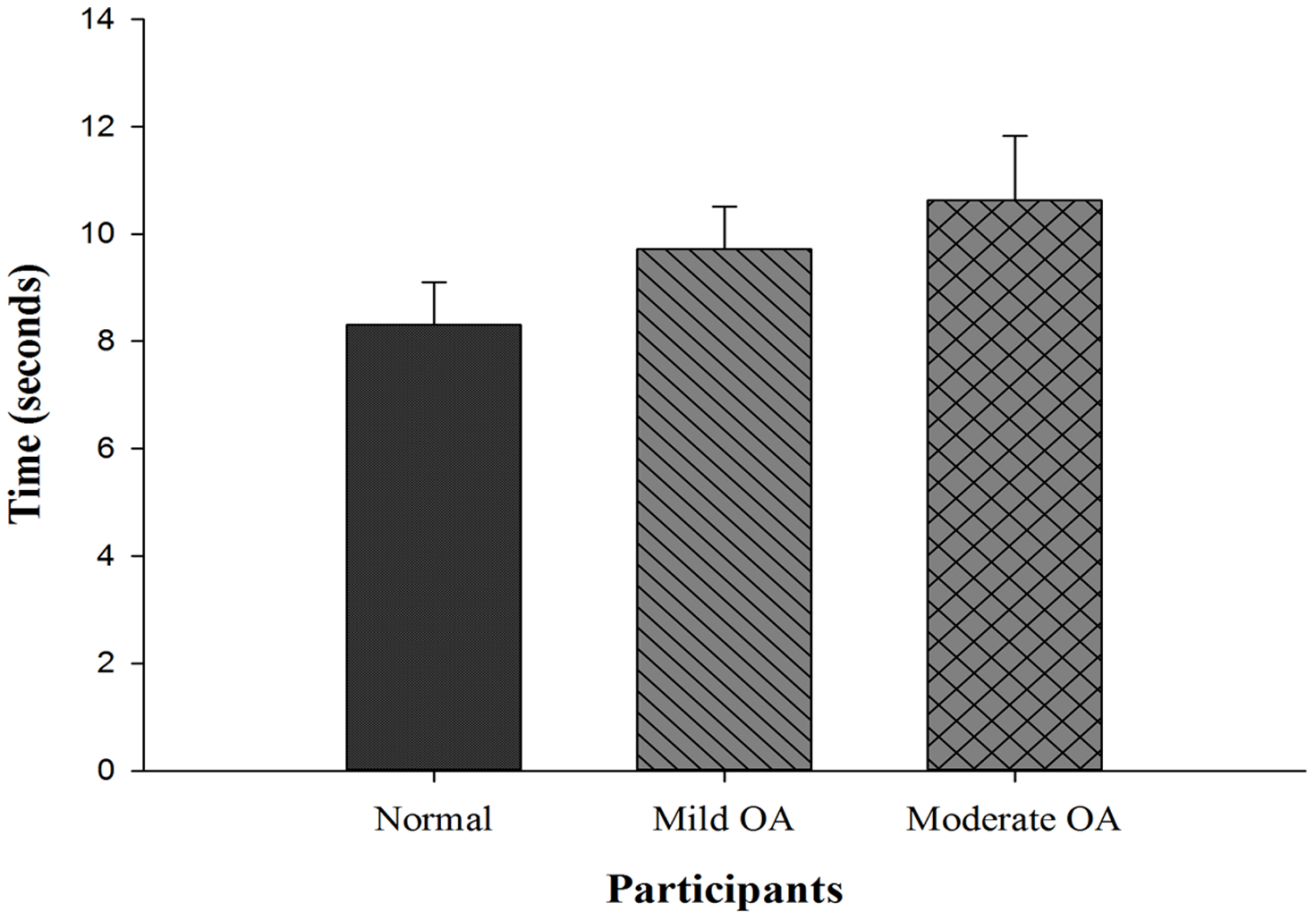
Timed Up and Go test.

Table 3 illustrates the mean and standard deviation results of balance and risk of fall in all the participants. It represents the overall, AP and ML scores (static and dynamic balance) in both function and defined conditions. Risk of fall results are presented only by overall score. Figure 1 presents the score of TUG (mean and SD) in three groups.

**Table 3:** Balance and risk of fall scores.

		Static		Dynamic level 8		Risk of fall level 8		Risk of fall level 6 to 2	
		Mean (SD)		Mean (SD)		Mean (SD)		Mean (SD)	
		Defined	Functional	Defined	Functional	Defined	Functional	Defined	Functional
Normal	Overall	0.65 (0.2)	0.56 (0.2)	1.15 (0.5)	1.11 (0.5)	0.99 (0.3)	0.81 (0.4)	1.70 (0.5)	1.50 (0.6)
	AP	0.44 (0.2)	0.45 (0.2)	0.85 (0.5)	0.70 (0.4)				
	ML	0.30 (0.1)	0.24 (0.1)	0.65 (0.3)	0.70 (0.4)				
Mild knee OA	Overall	0.80 (0.3)	0.64 (0.3)	1.70 (0.7)	1.70 (0.9)	1.50 (0.6)	1.30 (0.6)	2.70 (1.3)	2.30 (1.2)
	AP	0.65 (0.2)	0.44 (0.2)	1.10 (0.3)	1.17 (0.6)				
	ML	0.36 (0.2)	0.34 (0.2)	1.01 (0.6)	0.92 (0.6)				
Moderate knee OA	Overall	1.05 (0.8)	0.85 (0.4)	1.80 (0.7)	1.80 (0.7)	1.70 (0.6)	2.10 (1.1)	2.90 (1.1)	2.70 (1.2)
	AP	0.81 (0.6)	0.62 (0.35)	1.25 (0.5)	1.18 (0.5)				
	ML	0.49 (0.4)	0.46 (0.33)	1.15 (0.5)	1.06 (0.5)				

The results of one-way ANOVA are presented in table 4 (defined feet position) and table 5 (functional feet position and TUG) which only applied for overall scores. The results of this study showed that there is a significant difference between three groups in all the tests. The results of ANOVA are static overall-defined feet position: [F (2, 57) = 3.6, *p* = .035], static overall-functional feet position: [F (2, 57) = 4.5, *p* = .015], dynamic overall-defined feet position: [F (2, 57) = 6.2, *p* = .004], dynamic overall-functional feet position: [F (2, 57) = 5.1, *p* = .009], TUG: [F (2, 57) = 31.1, P = .000], risk of fall level 8-defined feet position: [F (2, 57) = 8.7, P = .001], risk of fall level 8-functional feet position: [F (2, 57) = 14.1, P = .000], risk of fall level 6 to 2 - defined feet position: [F (2, 57) = 6.0, P = .004] and risk of fall level 6 to 2-functional feet position: [F (2, 57) = 6.6, P = .003].

**Table 4:** ANOVA results for balance and risk of fall in defined feet position.

Dependent Variable	(I) Status	(J) Status	Mean Difference (I-J)	Sig
Static	Healthy	Grade II	−0.14	0.630
		Grade III	−0.40*	0.029
	Grade II	Healthy	0.14	0.630
		Grade III	−0.26	0.211
	Grade III	Healthy	0.40*	0.014
		Grade II	0.26	0.096
Dynamic	Healthy	Grade II	−0.54*	0.021
		Grade III	−0.64*	0.005
	Grade II	Healthy	0.54*	0.021
		Grade III	−0.10	0.866
	Grade III	Healthy	0.64*	0.005
		Grade II	0.10	0.866
Risk of fall (level 8)	Healthy	Grade II	−0.48*	0.016
		Grade III	−0.68*	0.000
	Grade II	Healthy	0.48*	0.016
		Grade III	−0.20	0.464
	Grade III	Healthy	0.68*	0.000
		Grade II	0.20	0.464
Risk of fall (level 6–2)	Healthy	Grade II	−1.01*	0.008
		Grade III	−0.93*	0.015
	Grade II	Healthy	1.01*	0.008
		Grade III	0.08	0.966
	Grade III	Healthy	0.93*	0.015
		Grade II	−0.08	0.966

*The mean difference is significant at the 0.05 level.

**Table 5:** ANOVA results for balance and risk of fall in functional feet position and TUG score.

Dependent Variable	(I) Status	(J) Status	Mean Difference (I-J)	Sig
Static	Healthy	Grade II	−0.08	0.701
		Grade III	−0.29*	0.014
	Grade II	Healthy	0.08	0.701
		Grade III	−0.21	0.096
	Grade III	Healthy	0.29*	0.014
		Grade II	0.21	0.096
Dynamic	Healthy	Grade II	−0.55*	0.042
		Grade III	−0.66*	0.012
	Grade II	Healthy	0.55*	0.042
		Grade III	−0.11	0.872
	Grade III	Healthy	0.66*	0.012
		Grade II	0.11	0.872
Risk of fall (level 8)	Healthy	Grade II	−0.54	0.075
		Grade III	−1.27*	0.000
	Grade II	Healthy	0.54	0.075
		Grade III	−0.74*	0.009
	Grade III	Healthy	1.27*	0.000
		Grade II	0.74*	0.009
Risk of fall (level 6–2)	Healthy	Grade II	−0.75	0.066
		Grade III	−1.17*	0.002
	Grade II	Healthy	0.75	0.066
		Grade III	−0.42	0.399
	Grade III	Healthy	1.17*	0.002
		Grade II	0.42	0.399
TUG	Healthy	Grade II	−1.43*	0.000
		Grade III	−2.35*	0.000
	Grade II	Healthy	1.43*	0.000
		Grade III	−0.92*	0.009
	Grade III	Healthy	2.35*	0.000
		Grade II	0.92*	0.009

*The mean difference is significant at the 0.05 level.

## Discussion

Evaluating balance can be important part of the rehabilitation programs. Balance disorders are growing public health problems due to their association with falls and fall-related injuries. Deficits in lower limb proprioception are associated with knee OA [26], [27] and thus may be postulated as a cause of impaired balance [4]. Decreased postural stability causes difficulties in performing activities of daily living which would affect the patient's quality of life [11].

Finding of this study revealed that there is a significant difference in balance and risk of fall between healthy, mild knee OA and moderate knee OA groups. To be precise, there was a significant difference in dynamic balance between healthy participants and knee OA patients (both feet positions). In contrast, for risk of fall the significant difference only exists in defined feet position. However, there was no significant difference between mild and moderate groups in the mentioned tests. Moreover, in static balance and risk of fall (functional feet position) difference existed only between healthy people and individuals with moderate knee OA. Interestingly, there was a significant difference in TUG scores between three groups, even between mild and moderate knee OA groups. In general, the findings of this study supported that individuals with bilateral knee OA had impaired balance compared to healthy controls, and this impairment was more pronounced in moderate knee OA patients.

Different assessments and devices are available for evaluation of balance and risk of fall. One of the methods which were used widely by researchers is force platform [1], [8], [18]. Some other methods were Swaymeter [4], Balance Performance Monitor [20] and Biodex stability system [11]. In this study Biodex Stability System was used. Although different assessments were recruited for assessing balance in knee OA patients, the results were in concordance with each other, and demonstrated that individuals with knee OA have impaired balance. In addition, our results corroborated previous findings.

Few studies assessed balance in knee OA patients by measuring postural sway [18]. Increased postural sway on the movement of centre of pressure (COP) is an evidence of impaired or deteriorated balance [18]. Individuals with knee OA display greater postural sway with both open and closed eyes condition [4], [8]. Greater postural sway was more obvious in individuals with radiographic knee OA than those without it [18]. Furthermore, Petrella et al. (2012) reported that elderly females with knee OA have an increased COP displacement in the anterior-posterior direction. Also, Hassan et al. (2001) assessed balance by measuring postural sway using Balance Performance Monitor and found that individuals with knee OA have increased anterior-posterior and medial-lateral sway in the standing position with closed eyes. The findings of all the mentioned studies supported each other and indicated postural control impairments in knee OA patients.

One of the explanatory factors for the variation of postural sway in individuals with knee OA is the severity of knee pain. Pain associated with osteoarthritis of the knees increased the propensity to trip on an obstacle, and the greater the pain is associated with greater risk of fall [28]. However, one study illustrated that knee pain is associated with poor balance in individuals with muscles weakness [29]. These findings underscore the importance of improving pain in individuals with knee OA. Moreover, the chronicity (longer time) of cartilage degeneration, as well as, muscle strength may influence balance and risk of fall in individuals with bilateral moderate and mild knee OA.

According to our knowledge, very few studies used BSS for assessing balance in individuals with knee OA, as well as, few considered only bilateral mild and moderate knee OA. A recent study which used BSS for balance assessment illustrated that individuals with knee OA displayed lower overall postural stability than controls [11], which means that postural stability is decreased in patients with knee OA. Our results support those of previous studies that reported impaired balance and higher risk of fall in knee OA patients compared to healthy controls. Importantly, this impairment was more noticeable in moderate knee OA patients.

## Conclusion

This study aimed to evaluate the static and dynamic balance and risk of fall in osteoarthritic knees. We found that balance (static and dynamic) in either feet position (defined or functional) is impaired in patients with bilateral knee osteoarthritis. The impairment seems to be more pronounced in moderate OA compared to mild OA. The impairment in balance is also coupled with higher risk of fall in the subjects.

Improving the postural stability of older adults with knee OA has become an important challenge. Establishing these data have implications in planning rehabilitation programs and will enable the practitioner to customise their rehabilitation strategies. Future studies need to determine the difference in postural stability and risk of fall between bilateral and unilateral knee OA; as well as, including severe knee OA group in their studies.
